# Supplementary material for: Fentanyl purity and overdose decline: A reexamination of geographic trends
Source: Sci Adv. 2026 Sep 16;12(38):eaei7742. doi: 10.1126/sciadv.aei7742 (PMC13580533; doi:10.1126/sciadv.aei7742)
Supplement: Supplementary file 1 [file sciadv.aei7742_sm.pdf]

Science Advances
AAAS

Supplementary Materials for

Fentanyl purity and overdose decline: A reexamination of geographic trends

Nabarun Dasgupta *et al.*

Corresponding author: Nabarun Dasgupta, nab@unc.edu

Sci. Adv. **12**, eaei7742 (2026)
DOI: 10.1126/sciadv.aei7742

This PDF file includes:

Supplementary Text
Figs. S1 to S3
Tables S1 to S4

Supplementary Text

S1: Fentanyl Purity Data Extraction Procedure

The fentanyl powder purity time series was obtained by digitizing the trend line from the published figure on page 23 of the 2025 DEA National Drug Threat Assessment (*71*), replicating the approach described by Vangelov et al. (*7*). Because the published figure was available only as a rendered image within an electronic document, direct numerical extraction was not possible, and a coordinate-based digitization procedure was employed.

Digitization Method

Monthly data points along the fentanyl powder purity trend line (red line, top panel of the DEA figure) were identified by visual inspection and plotted as anchor points on a vector path in Adobe Illustrator. Each anchor point was placed at the center of the plotted data marker or at the mid-line position for months where markers were not visually distinct. The path coordinates were then extracted programmatically using a custom JavaScript routine that read the anchor point positions from the Illustrator document object model and scaled them to the axis system of the original figure. The x-axis was mapped to monthly intervals (January 2019 through September 2024), and the y-axis was calibrated to the purity percentage scale using the labeled gridlines in the published figure.

Pixel-level anti-aliasing in the electronic PDF introduced minor positional ambiguity at each data point. To minimize systematic bias from aliasing artifacts, a conservative placement strategy was used: anchor points were positioned at the visually estimated centroid of the anti-aliased region rather than at any single edge, and borderline cases were resolved by erring toward the nearest labeled gridline. This approach may introduce small residual bias, the direction and magnitude of which are quantified below.

Replication Fidelity

The extracted time series was compared with the data published in the GitHub repository accompanying Vangelov et al. (*7*) using three complementary agreement statistics. Lin's concordance correlation coefficient, which simultaneously measures precision and accuracy between two sets of measurements, was 0.999, indicating near-perfect reproducibility. Bland–Altman analysis yielded a mean difference of +0.164 percentage points (our extraction marginally higher than Vangelov et al. (*7*)), with 95% limits of agreement spanning −0.182 to +0.509 percentage points. Pearson correlation between the two extractions was $r > 0.999$. These metrics collectively demonstrate negligible inter-extraction disagreement across all monthly observations (Fig. S1).

Coordinate Extraction Code

A custom JavaScript used for Illustrator coordinate extraction and axis calibration was written to extract data from the published figured. The script read anchor point positions from a selected path object in Adobe Illustrator 2026, applied linear interpolation to map pixel coordinates to the figure's axis units, and output a CSV of monthly purity values.

We note that the pill purity data from an adjacent panel in the same DEA figure were extracted but excluded from the primary analysis. No observable correlation existed between pill and powder purity trends (Fig. S2), the magnitude of pill purity variation was substantially lower, and irregular axis labeling (the vertical axis did not include zero) raised concerns about the validity of manual extraction for that series.

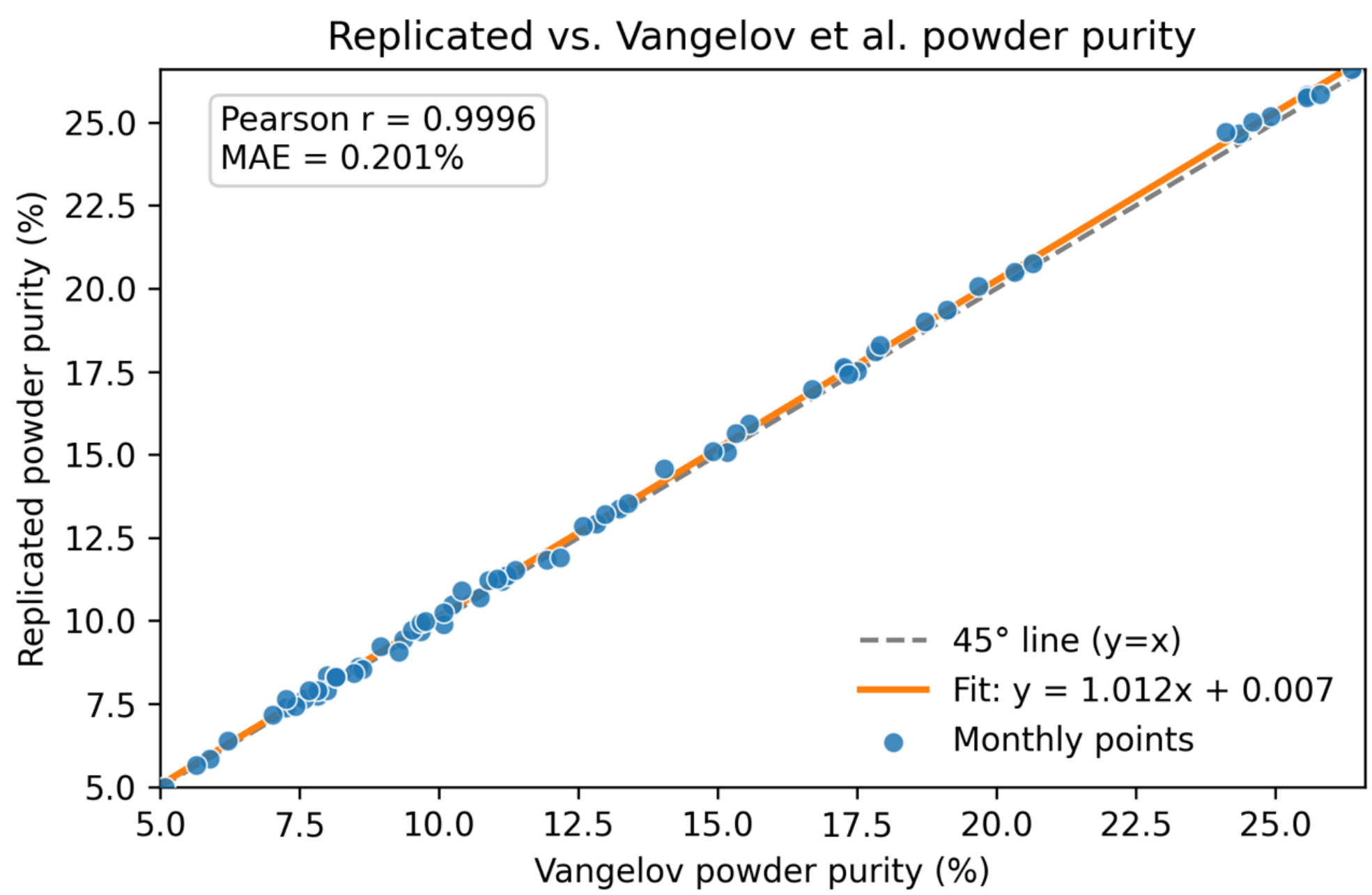

Fig. S1. Replication fidelity of fentanyl powder purity data extraction. Comparison of monthly fentanyl powder purity values extracted independently in the present study versus those published in the Vangelov et al. GitHub repository (*7*). Lin's concordance correlation coefficient = 0.999; Bland–Altman mean difference = +0.164 percentage points (limits of agreement: −0.182 to +0.509). The close agreement confirms that the digitization procedure reproduced the values reported by Vangelov et al. (*7*).

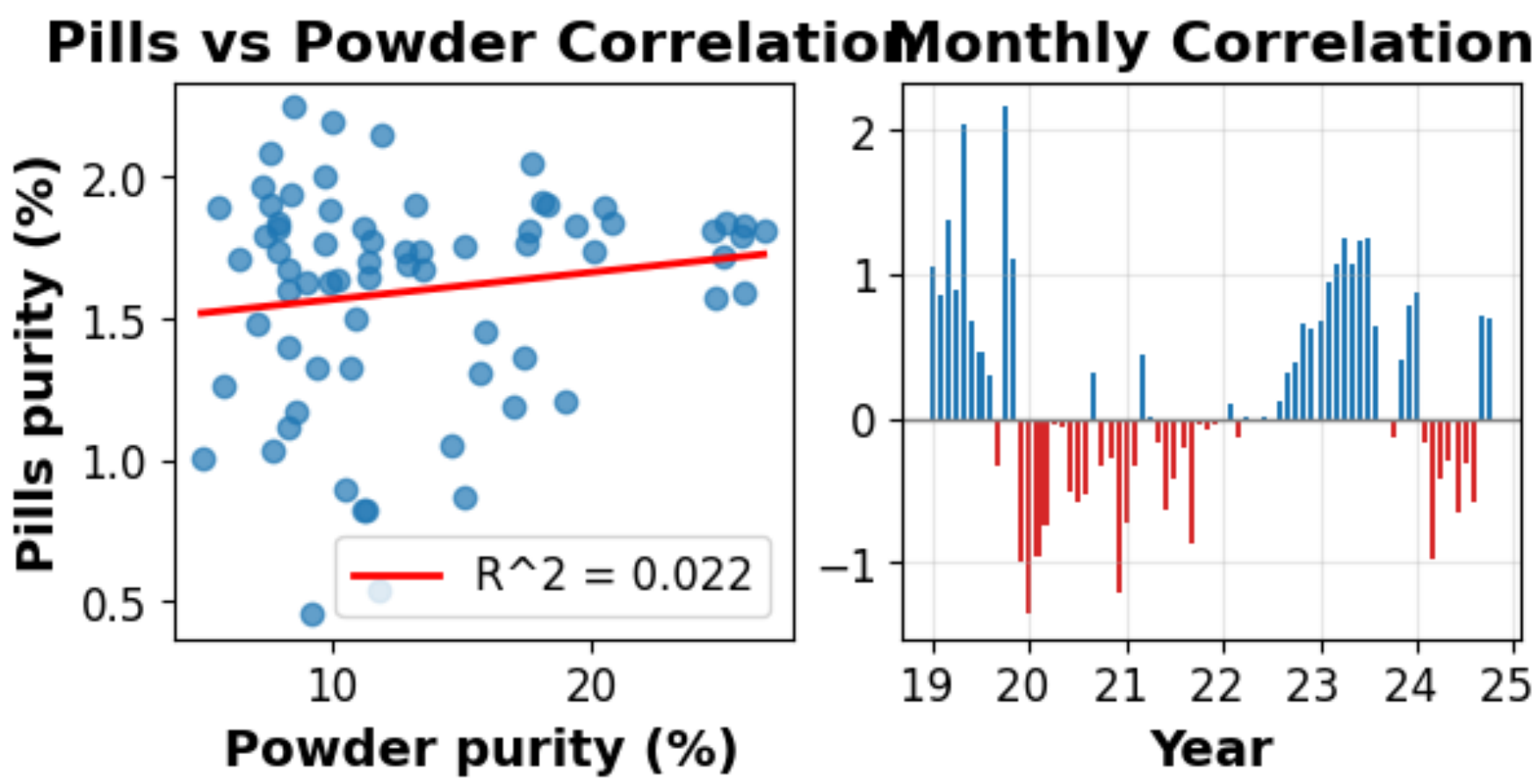

Fig. S2. Correlation between pill and powder fentanyl potency reported by the Drug Enforcement Agency (DEA). Scatterplot of monthly DEA-reported fentanyl pill potency (milligrams per tablet) versus powder purity (percent) across the study period. No meaningful correlation was observed between the two series, supporting the decision to restrict the primary analysis to powder purity data. Pill purity values are approximate given axis labeling discrepancies in the original DEA report.

S2: Negative Macroeconomic Controls

Rationale

A central methodological concern in interpreting the analysis by Vangelov et al. (*7*) is that the temporal correlation between nationally aggregated DEA fentanyl purity and overdose mortality may reflect shared post-COVID macrotrends rather than a pharmacologically meaningful supply-shock signal. Both fentanyl purity and overdose mortality followed a characteristic trajectory over 2019–2024: relative stability in the pre-pandemic period, disruption and surge during 2020–2021, peak plateau in 2022–2023, and concurrent decline thereafter. This trajectory is not unique to the drug supply or drug-related mortality—it characterizes a broad range of social and economic indicators affected by pandemic disruption and recovery.

When two non-stationary time series share a common trend, standard correlation statistics will detect a strong association even in the complete absence of any causal or mechanistic relationship. This phenomenon, known as spurious correlation, is well described in the time-series literature and motivates autocorrelation-adjustment procedures such as Box–Jenkins prewhitening prior to cross-correlation analysis (*29, 73*).

To illustrate the magnitude and direction of this confounding in the present context, we conducted a supplementary cross-correlation analysis using two economic indicators (the Consumer Price Index (CPI) and the University of Michigan Consumer Sentiment Index) that have no plausible direct pharmacological pathway to overdose mortality. If the unadjusted correlation between DEA purity and overdose mortality is meaningful, it should be substantially stronger than correlations with causally unrelated series. If similarly large correlations arise with multiple causally unrelated indicators, and if these correlations attenuate or reverse sign following autocorrelation adjustment, this provides strong evidence that the unadjusted purity–mortality association is driven primarily by shared post-COVID trend structure.

Methods

Data Sources and Variables

Monthly data spanning January 2019 through September 2024 were assembled from the sources listed in Table S1.

Table S1. Data sources, date ranges, and units for all variables included in the supplementary cross-correlation analysis.

Variable	Source	Date Range	Units
OD Mortality Rate	NCHS/CDC NVSS via OD Pulse	Jan 2019–Sep 2024	Deaths per 100,000 population/month
Consumer Price Index	U.S. Bureau of Labor Statistics (CPIAUCSL, via FRED)	Jan 2019–Sep 2024	12-month % growth rate
Michigan Consumer Sentiment	University of Michigan Survey	Jan 2019–Sep 2024	Index (1966:Q1 = 100)

NCHS = National Center for Health Statistics
CDC = Centers for Disease Control and Prevention
NVSS = National Vital Statistics System
OD = overdose
Q = calendar quarter

Analytical Approach

All analyses were conducted in R (version 4.3.3) using the *stats*, *forecast*, *ggplot2*, and *patchwork* packages. The analysis proceeded in three stages.

Stage 1: Visual Co-movement (Unadjusted Levels)

Pearson correlation coefficients were computed between the OD mortality rate (levels) and each economic indicator (levels) to quantify the unadjusted association. Series were Z-score normalized (mean = 0, SD = 1) and plotted together on a shared time axis. (Z-score standardization affects only the plotted scale and is provided to allow comparison between the two macroeconomic indicators on visually comparable graphs.) Pearson r was computed on the original unstandardized series.

Stage 2: Unadjusted Cross-Correlation Function

Cross-correlation functions (CCFs) were computed between OD mortality rate and each economic indicator across lags of −12 to +12 months, without autocorrelation adjustment. In the CCF, a positive lag k indicates that the input series at time t is associated with the output series at time $t+k$. The 95% confidence interval was computed as $\pm 1.96/\sqrt{n}$, where $n = 69$. (Confidence interval bound are approximate and do not adjust for degrees of freedom lost to differencing.) A CCF in which nearly all bars exceed the 95% CI across all lags is the expected signature of two autocorrelated, trending series and does not indicate genuine dynamic coupling.

Stage 3: Box-Jenkins Prewhitening

To remove autocorrelation structure from both series prior to CCF estimation, a standard Box-Jenkins prewhitening procedure was applied (*29, 73*). An ARIMA model was fitted to the OD

mortality rate series using automatic model selection via *auto.arima()* with Bayesian Information Criterion (BIC) and full model search (stepwise = FALSE). The selected model was ARIMA(0,2,1)(1,0,0)[12], indicating that the OD rate series required second-order differencing to achieve stationarity, with a moving-average term at lag 1 and a weak seasonal autoregressive component.

The residuals of this ARIMA fit constituted the prewhitened input series (α_t). The same AR/I/MA filter structure (order $p = 0$, $d = 2$, $q = 1$, seasonal $P = 1$, $D = 0$, $Q = 0$) was applied to the economic indicator series using the *Arima()* function with identical order constraints, yielding prewhitened output residuals (β_t). CCF was then computed between α_t and β_t across lags −12 to +12 months.

Table S2. Auto-ARIMA models selected by BIC for each series.

Series	Auto-ARIMA Model	BIC*	Interpretation
OD Mortality Rate	ARIMA(0,2,1)(1,0,0)[12]	-89.3	Second-order integrated; stochastic trend with time-varying slope, and weak seasonal autoregression
CPI (12-mo %)	ARIMA(1,1,0)(1,0,0)[12]	66.8	First-order integrated with AR(1) inertia and seasonal autoregression
Sentiment Index	ARIMA(0,1,0)	417.1	Random walk after first differencing; no exploitable autocorrelation

* Bayesian information criterion. Values are not comparable across the three series, since they are fitted to different data.
CPI = Consumer Price Index
OD = overdose
ARIMA = autoregressive integrated moving average
AR = autoregressive

Results

Unadjusted Associations

In unadjusted analysis of levels, the OD mortality rate exhibited large cross-correlations with both economic indicators (Table S3). The peak cross-correlation with CPI was +0.63, occurring at lag 0 and therefore identical to the contemporaneous Pearson correlation. The peak cross-correlation with the Consumer Sentiment Index was −0.73 at lag −1, with the sentiment series preceding mortality by one month.

The CCFs for both pairs showed the characteristic pattern of spurious correlation: bars significant across virtually all lags from −12 to +12 months, with no distinguishable peak lag structure (Fig. S3, Rows B1 and B2). This pattern is expected when two autocorrelated, trending series are compared without adjustment and does not constitute evidence of meaningful dynamic coupling.

Prewhitened Associations

After Box-Jenkins prewhitening, both associations changed substantially in magnitude and direction (Table S3; Fig. S3, Rows C1 and C2). The OD rate–CPI correlation reversed sign (prewhitened peak $r = -0.34$ at lag +1 month; unadjusted $r = +0.63$), indicating that the positive unadjusted correlation was attributable to shared trend structure. The direction reversal from positive-to-negative indicates that the unadjusted positive correlation was attributable to shared trend structure. The residual negative association should not be interpreted directionally; the peak at lag +1 would imply mortality innovations preceding CPI innovations, which has no plausible mechanism.

The OD rate–Sentiment correlation also reversed sign (prewhitened peak $r = +0.32$ at lag +3 months; unadjusted $r = -0.73$ at lag −1 month). The original negative association reflected the concurrent 2020–2022 deterioration and 2023–2024 recovery in both indicators. After prewhitening, the residual positive association at lag +3 months likely reflects a COVID-period artifact in which the overdose surge slightly lagged the initial lockdown shock that had already begun driving a sentiment recovery.

Table S3. Peak cross-correlation coefficients before and after Box-Jenkins prewhitening.

Series pair	Peak unadjusted r	Unadjusted lag	Prewhitened r	Prewhitened lag
OD Rate ~ CPI*	+0.63**	0	**−0.34**†**	+1
OD Rate ~ Sentiment	−0.73**	−1	**+0.32**†**	+3

* For OD Rate ~ CPI the peak occurred at lag 0, so this value equals the contemporaneous Pearson correlation.
** $p < 0.05$ ($|r| > 1.96/\sqrt{n} = 0.236$, $n = 69$)
† Sign reversal relative to unadjusted estimate.
CPI = Consumer Price Index
OD = overdose

Interpretation

The central finding of this supplementary analysis is that two economic indicators with no pharmacological mechanism linking them to overdose mortality produce peak unadjusted cross-correlations with the OD mortality rate ($|r| = 0.63$–0.73) that are comparable to or larger than the correlation reported by Vangelov et al. (*7*) between DEA fentanyl purity and overdose mortality of 0.37. Both correlations are attributable to the shared post-COVID trend: a broad rise in multiple social and economic stressors through 2021–2022, followed by concurrent amelioration from 2023 onward.

This does not imply that the purity–mortality relationship is necessarily spurious in every context. Rather, it demonstrates that in the presence of strong, shared non-stationarity of the kind observed across multiple social metrics in the 2019–2024 period, an unadjusted correlation

between any two trending series will appear large and statistically significant regardless of whether any genuine relationship exists. The appropriate analytic response—as implemented in the transfer function models reported in the main text—is to remove the autocorrelation structure from both the input (purity) and output (mortality) series before estimating dynamic associations, so that inference is based on month-to-month innovations rather than shared long-run trends.

Fentanyl purity also followed the same broad post-COVID arc (rising through 2022, declining thereafter), making it structurally identical, from a time-series perspective, to CPI and Consumer Sentiment. An analysis that does not account for this shared trend cannot distinguish a causal supply-shock signal from the kind of spurious co-movement illustrated here.

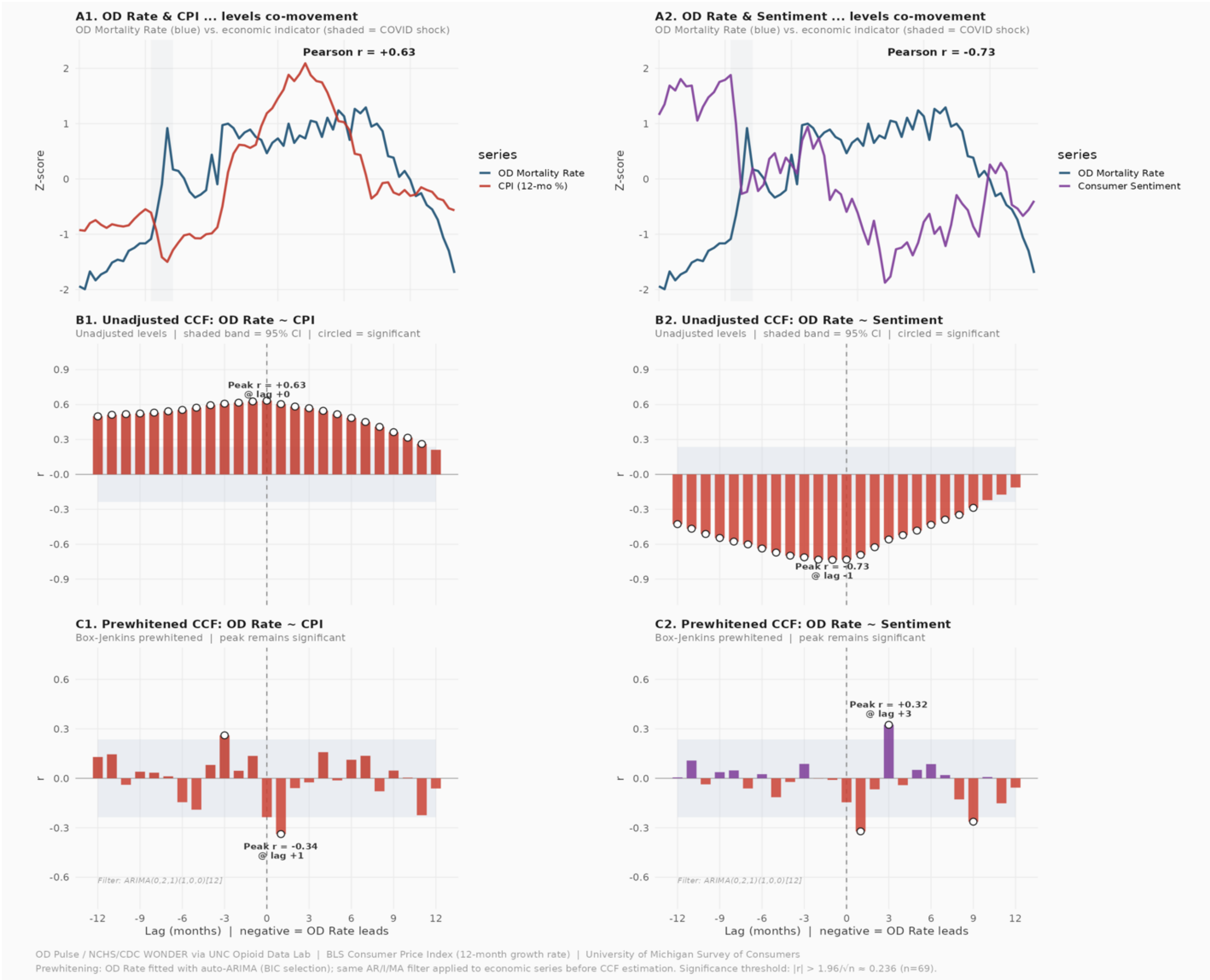

Fig. S3. Cross-correlation analysis of overdose mortality versus macroeconomic indicators.
Row A: Z-score normalized time series of OD mortality rate (red) overlaid with each economic indicator (blue) showing visual co-movement. Row B: Unadjusted cross-correlation functions (CCFs) across lags −12 to +12 months; virtually all bars exceed the 95% confidence interval (dashed lines), the expected signature of spurious correlation between autocorrelated trending series. Row C: Prewhitened CCFs after Box-Jenkins filtering; both associations reverse sign and attenuate markedly, demonstrating that the unadjusted correlations were artifacts of shared post-COVID trend structure rather than genuine dynamic coupling.

S3: Sampling Frame of 2023 DEA Fentanyl Powder Exhibits

DEA's Two Purity Data Sources: Regional Field Laboratories vs. the Fentanyl Profiling Program

DEA publicly reports fentanyl purity data from two distinct laboratory systems with different sampling frames and analytic purposes (*35*). Understanding their differences is essential for interpreting any purity time series derived from DEA data.

The first source is the network of 10 DEA regional field laboratories distributed across the United States, where routine law enforcement seizures—ostensibly at or near the street retail level—are identified and quantitated within the context of criminal prosecution. In CY [calendar year] 2023, the AIDD state-level table (*35*, page 4) sums to 7,737 exhibits ("samples" used interchangeably in report); a summary figure on page 6 (*35*) reports 7,857. State-level counts are used throughout; the 120-exhibit difference (1.6%) does not materially affect regional proportions. The geographic distribution of exhibits across states, presented in Table S4 below, is drawn from this source. For CY 2023, the average powder purity reported by these regional field laboratories was 19.7% (*35*, page 3).

The second source is the DEA's Fentanyl Profiling Program (FPP), housed within the Special Testing and Research Laboratory (SFL1) in the Office of Forensic Sciences. FPP conducts detailed forensic chemical analysis on a smaller, intentionally selected subset of seizures, including synthetic route classification, adulterant profiling, and purity quantitation for scientific and intelligence purposes.

In recent years, FPP shifted its sampling focus toward higher-volume ("wholesale") seizures. DEA explicitly stated:

> *"The significant increase in purity in CY [calendar year] 2023 is likely due to a change in the FPP sampling plan, which changed to focus more on wholesale level seizures. These larger seizures generally have a higher purity as they are not yet significantly adulterated domestically." (35, page 9)*

The report further specified the timing:

> *"In January 2023, FPP focused efforts on seized samples weighing greater than 800 g. In July 2023, new sampling guidance was released to the DEA laboratory system advising the submission of samples containing fentanyl, fentanyl-related compounds, and precursor chemicals when the net weight is greater than or equal to 800 g." (35, page 19)*

For CY 2023, the average powder purity reported by FPP was 33.5%, approximately 70% higher than the 19.7% average from the regional field laboratories (*35*, pages 3 and 9). This discrepancy is a direct and expected consequence of the 800-gram minimum weight threshold, which

systematically selects for wholesale-level seizures that have not yet been adulterated for retail distribution.

The composition of the purity time series published in the 2025 National Drug Threat Assessment (*71*)—the series digitized and analyzed by Vangelov et al. (*7*)—was not specified in that report. It is unclear whether this series includes FPP wholesale samples, regional field lab retail samples, or both, or whether the two sources are statistically weighted. The July 2023 sampling guidance was also provided to the regional DEA field laboratories (*35*), further complicating interpretation of any aggregate purity estimate from this period. Discrepancies between the Vangelov et al. (*7*) purity values and other published DEA reports (discussed in the main text) suggest that higher-purity wholesale FPP samples were comingled with routinely collected retail-level field laboratory samples, potentially inflating the apparent purity peak in 2023.

Geographic Distribution of Regional Field Laboratory Exhibits

Table S4 presents the geographic distribution of fentanyl powder exhibits analyzed by the DEA regional field laboratories (not the FPP) in CY 2023. These data are drawn from the AIDD powders table on page 4 of the Annual Fentanyl Report CY 2023 (*35*). Only powder exhibits (crystalline, powder, and rock-like forms; $n = 7{,}737$) are included; tablet exhibits ($n = 5{,}958$) are reported separately by DEA and excluded here. States are grouped by U.S. Census Region to correspond with the region-stratified transfer function models in the main text.

Out of 7,737 total fentanyl powder exhibits, the South accounted for 3,370 (43.6%), followed by the Northeast with 1,987 (25.7%), the Midwest with 1,263 (16.3%), and the West with 1,117 (14.4%). Three states, Florida ($n = 751$), California ($n = 657$), and New York ($n = 498$), together contributed 24.6% of all powder exhibits nationally. Within each region, sample counts were heavily concentrated: Florida and West Virginia collectively represented 35.9% of the South; Missouri and Illinois represented 50.8% of the Midwest; California alone represented 58.8% of the West; and New York and Massachusetts represented 42.1% of the Northeast.

These distributions should be considered when interpreting the "national" fentanyl purity series. Net seizure weight does not correspond proportionally to exhibit counts; for example, Tennessee contributed 219 exhibits representing 223.2 kg, while West Virginia contributed 459 exhibits representing only 12.3 kg, reflecting differences in seizure size distributions across jurisdictions.

Table S4. Fentanyl powder exhibits analyzed by DEA regional field laboratories, by state and US Census Region, 2023. Source: DEA *Annual Fentanyl Report CY 2023* (PRB# 2025-017), AIDD data, page 4 (powders column) (*35*).

State	Census Region	Number of Exhibits	% National	% Region	Net Weight (kg)	% National Weight	% Regional Weight
CT	Northeast	301	3.9	15.1	28.4	1.3	5.8
MA		339	4.4	17.1	84.3	3.8	17.2
ME		71	0.9	3.6	8.4	0.4	1.7
NH		119	1.5	6.0	13.6	0.6	2.8
NJ		255	3.3	12.8	60.0	2.7	12.2
NY		498	6.4	25.1	227.7	10.2	46.4
PA		293	3.8	14.7	48.6	2.2	9.9
RI		49	0.6	2.5	16.9	0.8	3.4
VT		62	0.8	3.1	2.6	0.1	0.5
	**Northeast Subtotal**	**1987**	**25.7**	**100.0**	**490.5**	**22.0**	**100.0**
IA	Midwest	15	0.2	1.2	0.5	0.0	0.2
IL		229	3.0	18.1	107.1	4.8	35.0
IN		139	1.8	11.0	30.9	1.4	10.1
KS		8	0.1	0.6	11.9	0.5	3.9
MI		167	2.2	13.2	74.3	3.3	24.3
MN		2	0.0	0.2	0.04	0.0	0.0
MO		413	5.3	32.7	60.3	2.7	19.7
ND		4	0.1	0.3	0.1	0.0	0.0
NE		15	0.2	1.2	0.1	0.0	0.0
OH		158	2.0	12.5	14.0	0.6	4.6
SD		1	0.0	0.1	0.01	0.0	0.0
WI		112	1.4	8.9	7.0	0.3	2.3
	**Midwest Subtotal**	**1263**	**16.3**	**100.0**	**306.3**	**13.7**	**100.0**
AL	South	96	1.2	2.8	47.1	2.1	5.6
AR		73	0.9	2.2	13.5	0.6	1.6

DC		179	2.3	5.3	4.6	0.2	0.5
DE		78	1.0	2.3	12.3	0.6	1.5
FL		751	9.7	22.3	167.2	7.5	19.9
GA		154	2.0	4.6	25.5	1.1	3.0
KY		209	2.7	6.2	31.4	1.4	3.7
LA		54	0.7	1.6	11.7	0.5	1.4
MD		129	1.7	3.8	14.6	0.7	1.7
MS		33	0.4	1.0	3.6	0.2	0.4
NC		296	3.8	8.8	59.9	2.7	7.1
OK		115	1.5	3.4	27.0	1.2	3.2
PR		95	1.2	2.8	6.9	0.3	0.8
SC		88	1.1	2.6	48.0	2.2	5.7
TN		219	2.8	6.5	223.2	10.0	26.5
TX		215	2.8	6.4	109.2	4.9	13.0
VA		127	1.6	3.8	23.1	1.0	2.7
WV		459	5.9	13.6	12.3	0.6	1.5
	**South Subtotal**	**3370**	**43.6**	**100.0**	**841.1**	**37.7**	**100.0**
AK	West	18	0.2	1.6	3.7	0.2	0.6
AZ		67	0.9	6.0	66.0	3.0	11.2
CA		657	8.5	58.8	416.1	18.7	70.3
CO		27	0.3	2.4	8.6	0.4	1.5
HI		23	0.3	2.1	3.4	0.2	0.6
ID		3	0.0	0.3	0.03	0.0	0.0
MT		38	0.5	3.4	1.4	0.1	0.2
NM		9	0.1	0.8	5.1	0.2	0.9
NV		19	0.2	1.7	1.7	0.1	0.3
OR		109	1.4	9.8	50.1	2.2	8.5
UT		1	0.0	0.1	0.01	0.0	0.0
WA		146	1.9	13.1	35.6	1.6	6.0

	**West Subtotal**	**1117**	**14.4**	**100.0**	**591.7**	**26.5**	**100.0**
	**National Total**	**7737**	**100.0**	**—**	**2229.6**	**100.0**	**—**

Notes: Exhibits reflect powder forms only (crystalline, powder, and rock-like); tablet exhibits (*n* = 5,958) are excluded. Percentages may not sum to exactly 100.0% due to rounding. Census Region assignments follow US Census Bureau definitions. DC and PR are classified within the South following DEA field office jurisdictional convention. The DEA regional laboratory system comprises 10 laboratories distributed across the United States; exhibit counts per state reflect the volume of drug evidence submitted to and analyzed by the laboratory serving each jurisdiction, not a statistically representative sample of the illicit drug supply. The regional field laboratory average powder purity for CY 2023 was 19.7%, compared to 33.5% for the intentionally selected FPP wholesale samples (*35*). kg = kilograms
